# Prevalence of and risk factors for binge eating behaviour in 6930 adults starting a weight loss or maintenance programme

**DOI:** 10.1017/S1368980015001068

**Published:** 2015-05-11

**Authors:** Simona Bertoli, Alessandro Leone, Veronica Ponissi, Giorgio Bedogni, Valentina Beggio, Maria Grazia Strepparava, Alberto Battezzati

**Affiliations:** 1 International Center for the Assessment of Nutritional Status (ICANS), Department of Food, Environmental and Nutritional Sciences (DEFENS), University of Milan, Via Botticelli 21, 20133 Milan, Italy; 2 Clinical Epidemiology Unit, Liver Research Center, Basovizza, Trieste, Italy; 3 Department of Health Sciences, School of Medicine, University of Milan–Bicocca, Milan, Italy

**Keywords:** Epidemiology, Cross-sectional study, Obesity, Weight loss, Binge eating, Anxiety, Depression

## Abstract

**Objective:**

Conflicting data are available on the prevalence of binge eating behaviour (BE) in individuals seeking to lose or maintain weight. The present study aimed to estimate the prevalence of and the risk factors for BE in a large sample of men and women starting a weight loss or maintenance programme.

**Design:**

Cross-sectional study. BE was defined as a Binge Eating Scale (BES) score ≥18. The State-Trait Anxiety Inventory and the Italian Depression Questionnaire were used to assess anxiety and depression. Besides sex, age and BMI, marital status, educational level, smoking and physical activity were evaluated as potential risk factors for BE. Uni- and multivariable Poisson working regression models were used to estimate prevalence ratios (PR) and marginal probabilities.

**Setting:**

Nutritional research centre.

**Subjects:**

Adults (*n* 6930; 72 % women) with a median age of 46 years (range 18–81 years) were consecutively studied.

**Results:**

BE prevalence in the pooled sample was 17 %. At multivariable analysis, being a woman (PR=2·70), smoking (PR=1·15) and increasing BMI (PR=1·05 for 1 kg/m^2^ increase) were risk factors for BE. On the contrary, being older (PR=0·99 for 1-year increase), performing physical activity (PR=0·89) and being married (PR=0·88) were protective factors for BE. Anxiety and depression were more common in subjects with BE.

**Conclusions:**

BE is common in individuals seeking to lose or maintain weight. The prevalence of BE is higher in young obese women. However, BE is present also in men, elders and normal-weight subjects.

Binge eating behaviour (BE) is characterized by: (i) eating, in a discrete period of time, an amount of food that is definitely larger than what most people would eat in a similar period of time under similar circumstances; and (ii) a sense of lack of control over eating^(^ ^1^ ^)^. BE is the primary diagnostic criterion of several disorders, i.e. binge eating disorder, bulimia nervosa, some subtypes of anorexia nervosa, and eating disorder not otherwise specified^(^ ^1^ ^)^.

BE is associated with excess energy intake, contributing to obesity and its complications^(^ ^2^ ^)^. Diet therapy is the first-line treatment for obesity but about three-quarters of obese people under dietary treatment regain weight within one year^(^ ^3^ ^)^. BE is associated with weight regain^(^ ^4^ ^–^ ^6^ ^)^, failed weight loss^(^ ^7^ ^–^ ^9^ ^)^ and anxiety and depression^(^ ^10^ ^,^ ^11^ ^)^. Early identification of BE is thus important in persons seeking to lose or maintain weight^(^ ^12^ ^)^.

The prevalence of BE among individuals participating in weight-loss programmes has been reported to vary from 23 to 55 %^(^ ^13^ ^)^, but recent studies report lower estimates (17–19 %)^(^ ^14^ ^,^ ^15^ ^)^. The high variability of these estimates is partly explained by the heterogeneity of the study populations, the generally low sample sizes and the use of different methods to detect BE. Moreover, few and conflicting data are available on the association of BE with sex, age and nutritional status as most of the available studies have been performed in young obese women^(^ ^5^ ^,^ ^7^ ^,^ ^15^ ^–^ ^17^ ^)^.

The present study, performed in a large sample of participants to a weight loss or maintenance programme, aimed to: (i) estimate the prevalence of BE; and (ii) test whether sex, age, BMI, educational level, smoking, physical activity and marital status are risk factors for BE.

## Methods

### Study design

We performed a cross-sectional study on 7524 consecutive adults who self-referred to the International Center for the Assessment of Nutritional Status (ICANS, University of Milan) from July 2008 to April 2014, in order to participate to a structured weight-loss or weight-maintenance programme. On the same day, they underwent a clinical examination, an anthropometric assessment and a structured interview by a trained dietitian, in order to obtain information about marital status, educational level, smoking and structured physical activity. The latter was investigated by asking subjects the following questions: ‘Do you practise any structured physical activity?’ and ‘How many hours per week do you spend on this activity?’ Individuals who devoted ≥2 h/week to any structured physical activity were considered as active. Moreover, all subjects filled in four questionnaires to evaluate the presence of BE and anxious and depressive symptoms. In order to be eligible for the study, the subjects had to be at least 18 years old and free of major organ disease (e.g. heart failure, cancer, neurological disease).

### Anthropometric assessment

Anthropometric measurements were performed following international guidelines^(^ ^18^ ^)^. Body weight was measured to the nearest 100 g using a SECA 700 scale and height was measured to the nearest 0·1 cm using a SECA 217 vertical stadiometer (SECA, Hamburg, Germany). BMI was calculated as [weight (kg)]/[height (m)]^2^ and classified according to the WHO cut-offs (BMI=18·5–24·9 kg/m^2^, normal weight; BMI=25·0–29·9 kg/m^2^, overweight; BMI=30·0–34·9 kg/m^2^, obesity class 1; BMI=35·0–39·9 kg/m^2^, obesity class 2; and BMI≥40· kg/m^2^, obesity class 3)^(^ ^19^ ^)^.

### Psychological assessment

Eating behaviour was evaluated using the Italian version of the Binge Eating Scale (BES)^(^ ^20^ ^,^ ^21^ ^)^. BE was defined as a BES score ≥18^(^ ^8^ ^)^. Anxious symptoms were evaluated using the Italian version of Spielberg *et al*.’s State-Trait Anxiety Inventory (STAI)^(^ ^22^ ^)^. Individuals with a STAI-X1 (state anxiety) score ≥99th percentile (raw score ≥65 for men and ≥71 for women) were excluded from the study. Anxiety was defined as a STAI-X2 (trait anxiety) score ≥95th percentile (raw score ≥56 for men and ≥62 for women)^(^ ^23^ ^)^. Depression was evaluated using the Italian Depression Questionnaire (DQ) and defined as a DQ score ≥95th percentile (raw score ≥10 for men and ≥15 for women)^(^ ^23^ ^,^ ^24^ ^)^. Questionnaires were considered invalid when more of 10 % of items were missing^(^ ^23^ ^)^.

### Statistical analysis

Most continuous variables had non-Gaussian distributions and all are reported as 25th, 50th and 75th percentiles. Discrete variables are reported as counts and percentages. A Poisson working regression model (PWRM) with robust 95 % confidence interval was used to estimate prevalence and prevalence ratios (PR)^(^ ^25^ ^,^ ^26^ ^)^. A PWRM was used because a binomial regression model (BRM) failed to converge for some of the regressions of interest^(^ ^25^ ^,^ ^26^ ^)^. Expectedly, the PR estimated by the PWRM and by the BRM were similar in all cases where both could computed^(^ ^25^ ^,^ ^26^ ^)^. Uni- and multivariable PWRM were used to evaluate the associations of BE with sex, age, BMI, educational level, smoking status, physical activity and marital status. Univariable PWRM were used to model the association of anxiety and depression with sex, age and BMI. The outcome variables of all models (BE, anxiety, depression) were discrete (0=no; 1=yes). The covariates were coded as follows: (i) sex (discrete, 0=male; 1=female); (ii) age (continuous, years); (iii) BMI (continuous, kg/m^2^); (iv) educational level (discrete, 0=<high school; 1=≥high school); (v) smoking status (discrete, 0=no; 1=yes); (vi) physical activity (discrete, 0=no; 1=yes); and (vii) marital status (discrete, 0=unmarried; 1=married). Prevalence ratios and marginal probabilities were calculated from PWRM^(^ ^27^ ^,^ ^28^ ^)^. Univariable and multivariable fractional polynomials were used to test whether the relationships of continuous predictors with the outcomes were non-linear^(^ ^29^ ^)^. As there was a statistically significant but practically irrelevant improvement in model fit following an inverse transformation of BMI in the multivariable PWRM having BE as outcome, BMI was kept linear with the benefit of making the relationships more understandable to a clinical audience^(^ ^30^ ^)^.

## Results

### Study population

Out of 7524 eligible subjects, seventy-one had a STAI-XI score ≥99th percentile and were excluded from analysis as specified by the study protocol. Moreover, 523 subjects had at least one invalid questionnaire. In particular, 396 did not complete the BES questionnaire, seventy-six had missing answers in the STAI-X2 questionnaire and fifty-one had missing answers in the DQ. Thus data from 6930 subjects were available for analysis and their measurements are given in Table 1. The participants were mostly women (72 %) and aged 18–81 years (median age=46 years).

**Table 1:** Anthropometric and psychological measurements of the study subjects; men and women (median age 46 years) starting a weight loss or maintenance programme, Milan, Italy, July 2008 to April 2014

	Sex								
	Men (*n* 1932)			Women (*n* 4998)			Total (*n* 6930)		
	P_25_	P_50_	P_75_	P_25_	P_50_	P_75_	P_25_	P_50_	P_75_
Age (years)	38	47	56	36	45	55	37	46	55
Weight (kg)	83·8	93·1	104·7	64·8	73·0	82·9	67·7	78·1	91·1
Height (m)	1·71	1·75	1·79	1·57	1·62	1·66	1·59	1·64	1·71
BMI (kg/m^2^)	27·8	30·5	33·7	24·8	27·9	31·8	25·5	28·6	32·5
STAI-X1	30	34	38	32	37	43	32	36	42
STAI-X1 (SDS)	−0·83	−0·46	0·20	−0·76	−0·30	0·38	−0·79	−0·35	0·36
STAI-X2	30	35	41	34	40	48	33	39	46
STAI-X2 (SDS)	−0·82	−0·30	0·37	−0·75	−0·12	0·58	−0·78	−0·20	0·53
DQ	1	2	5	2	4	8	1	4	7
DQ (SDS)	−0·88	−0·57	0·24	−0·84	−0·35	0·30	−0·88	−0·39	0·29
BES	4	7	12	5	10	16	5	9	15

*n*, number of subjects; P_50_, 50th percentile; P_25,_ 25th percentile; P_75_, 75th percentile; STAI-X1, State-Trait Anxiety Inventory (state anxiety) score; SDS, standard deviation score; STAI-X2, State-Trait Anxiety Inventory (trait anxiety) score; DQ, Italian Depression Questionnaire score; BES, Binge Eating Scale score.

Table 2 reports the anthropometric status, marital status, educational level, smoking status, physical activity, and the frequency of BE, anxiety and depression in all subjects as well as stratified by sex.

**Table 2:** Anthropometric, lifestyle and psychological features of the study subjects; men and women (median age 46 years) starting a weight loss or maintenance programme, Milan, Italy, July 2008 to April 2014

	Sex					
	Men (*n* 1932)		Women (*n* 4998)		Total (*n* 6930)	
	*n*	%	*n*	%	*n*	%
Age (decade)						
18–19 years	17	0·9	71	1·4	88	1·3
20–29 years	139	7·2	537	10·7	676	9·8
30–39 years	385	19·9	1089	21·8	1474	21·3
40–49 years	586	30·3	1403	28·1	1989	28·7
50–59 years	429	22·2	1112	22·2	1541	22·2
60–69 years	301	15·6	639	12·8	940	13·6
70–79 years	75	3·9	146	2·9	221	3·2
≥80 years	0	0·0	1	0·0	1	0·0
Total	1932	100·0	4998	100·0	6930	100·0
BMI class						
Normal weight	144	7·5	1294	25·9	1438	20·8
Overweight	726	37·6	1954	39·1	2680	38·7
Obesity class 1	697	36·1	1119	22·4	1816	26·2
Obesity class 2	269	13·9	427	8·5	696	10·0
Obesity class 3	96	5·0	204	4·1	300	4·3
Total	1932	100·0	4998	100·0	6930	100·0
Married						
No	782	40·5	2418	48·4	3200	46·2
Yes	1150	59·5	2580	51·6	3730	53·8
Total	1932	100·0	4998	100·0	6930	100·0
High school or higher degree						
No	1142	59·1	3197	64·0	4339	62·6
Yes	790	40·9	1801	36·0	2591	37·4
Total	1932	100·0	4998	100·0	6930	100·0
Current smoking						
No	1256	65·0	3788	75·8	5044	72·8
Yes	676	35·0	1210	24·2	1886	27·2
Total	1932	100·0	4998	100·0	6930	100·0
Structured physical activity						
No	1077	55·7	2999	60·0	4076	58·8
Yes	855	44·3	1999	40·0	2854	41·2
Total	1932	100·0	4998	100·0	6930	100·0
Binge eating behaviour (BES≥18)						
No	1775	91·9	3986	79·8	5761	83·1
Yes	157	8·1	1012	20·2	1169	16·9
Total	1932	100·0	4998	100·0	6930	100·0
Anxiety (STAI-X1≥95th percentile)*						
No	1862	96·4	4837	96·8	6699	96·7
Yes	70	3·6	161	3·2	231	3·3
Total	1932	100·0	4998	100·0	6930	100·0
Anxiety (STAI-X2≥95th percentile)						
No	1859	96·2	4801	96·1	6660	96·1
Yes	73	3·8	197	3·9	270	3·9
Total	1932	100·0	4998	100·0	6930	100·0
Depression (DQ≥95th percentile)						
No	1845	95·5	4869	97·4	6714	96·9
Yes	87	4·5	129	2·6	216	3·1
Total	1932	100·0	4998	100·0	6930	100·0

*n*, number of subjects; %, percentage of subjects; BES, Binge Eating Scale score; STAI-X1, State-Trait Anxiety Inventory (state anxiety) score; STAI-X2, State-Trait Anxiety Inventory (trait anxiety) score; DQ, Italian Depression Questionnaire score.

* Subjects with STAI-X1≥99th percentile were excluded from the study.

### Prevalence of and risk factors for binge eating

The prevalence of BE in the pooled sample was 17 % (95 % CI 16 %, 18 %). Such prevalence was higher in women compared with men (PR=2·49, 95 % CI 2·12, 2·92, *P*<0·001; corresponding to probabilities of 20 % *v*. 8 %), decreased with increasing age (PR=0·99, 95 % CI 0·98, 0·99 for each 1-year increase, *P*<0·001) and increased with increasing BMI (PR=1·03, 95 % CI 1·03, 1·04 for each 1-kg/m^2^ increase, *P*<0·001; univariable PWRM).

Table 3 reports the PR obtained from the multivariable PWRM. As educational level was not associated with BE prevalence (model 1 of Table 3), it was removed from the final model (model 2 of Table 3). The PR of the remaining covariates changed very little from model 2 to model 1. In the final model (model 2 of Table 3), age (PR=0·99 for each 1-year increase), physical activity (PR=0·89) and being married (PR=0·88) were inversely associated with BE prevalence. On the contrary, being a woman (PR=2·70) and smoking (PR=1·15) were directly associated with the prevalence of BE. It should be noted that the 95 % CI of the PR associated with smoking (1·02, 1·29), physical activity (0·80, 0·99) and marital status (0·79, 0·99) were wide, suggesting that larger samples of subjects are needed to estimate these effects with acceptable precision.

**Table 3:** Predictors of binge eating prevalence among men and women (*n* 6930; median age 46 years) starting a weight loss or maintenance programme, Milan, Italy, July 2008 to April 2014

	Model 1		Model 2	
	PR	95 % CI	PR	95 % CI
Female	2·68***	2·29, 3·14	2·70***	2·30, 3·16
Age (years)	0·99***	0·98, 0·99	0·99***	0·98, 0·99
BMI (kg/m^2^)	1·05***	1·04, 1·06	1·05***	1·04, 1·06
High school or higher degree	0·92	0·82, 1·03	—	
Smoker	1·14*	1·02, 1·29	1·15*	1·02, 1·29
Structured physical activity	0·89*	0·80, 0·99	0·89*	0·80, 0·99
Married	0·88*	0·79, 0·99	0·88*	0·79, 0·99
*n*	6930		6930	

Values are prevalence ratios (PR) with robust 95 % confidence intervals obtained from a multivariable Poisson working model; *n*, number of subjects. *P<0·05, **P<0·001.

**Fig. 1:**
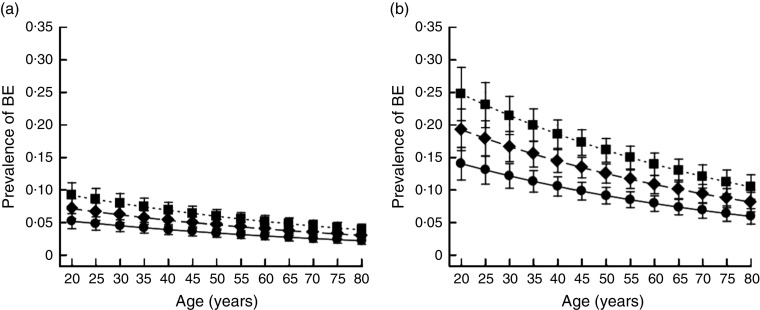
Prevalence of binge eating (BE) as a function of sex (a, men; b, women), age and BMI (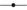, 18·5 kg/m^2^; 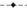, 24·9 kg/m^2^; 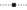, 30·0 kg/m^2^) among 6930 subjects (median age 46 years) starting a weight loss or maintenance programme, Milan, Italy, July 2008 to April 2014. Values are point estimates of marginal probabilities, with 95 % confidence intervals indicated by vertical bars, estimated for married, non-smoking and physically active individuals

Figure 1 plots the prevalence of BE estimated from model 2 of Table 3 as a function of age and BMI in married and non-smoking men and women. It can be seen that the prevalence of BE is higher in females across all ages and BMI levels.

### Prevalence of anxiety

The prevalence of anxiety in the pooled sample was 4 % (95 % CI 3 %, 4 %). Such prevalence was similar for women *v*. men (PR=1·04, 95 % CI 0·80, 1·36, *P*=0·75) but increased with increasing age (PR=1·03, 95 % CI 1·01, 1·04, *P*<0·001 for 1-year increase) and BMI (PR=1·05, 95 % CI 1·03, 1·07, *P*<0·001 for 1-kg/m^2^ increase; univariable PWRM). Expectedly, the prevalence of anxiety was higher in subjects with than in those without BE (PR=4·20, 95 % CI 3·40, 5·40, *P*<0·001; corresponding to probabilities of 11 % *v*. 3 %).

### Prevalence of depression

The prevalence of depression in the pooled sample was 3 % (95 % CI 3 %, 4 %). It was lower in women compared with men (PR=0·57, 95 % CI 0·43, 0·75, *P*<0·001), did not change with age (PR=0·99, 95 % CI 0·99, 1·00, *P*=0·24 for 1-year increase) and increased with BMI (PR=1·06, 95 % CI 1·04, 1·08, *P*<0·001 for 1-kg/m^2^ increase; univariable PWRM). The prevalence of depression was higher subjects with than in those without BE (PR=5·00, 95 % CI 3·90, 6·50, *P*<0·001; corresponding to probabilities of 9 % *v*. 2 %).

Lastly, the prevalence of both anxious and depressive symptoms was higher in BE than in non-BE subjects (5 %, 95 % CI 3 %, 7 % *v*. 1 %, 95 % CI 0 %, 1 %, *P*<0·001; univariable PWRM).

## Discussion

To our knowledge, the present study is the largest performed so far to estimate the prevalence of and the risk factors for BE in men and women enrolling into a weight loss or maintenance programme and the first study to systematically evaluate potential risk factors for BE besides sex, age and BMI.

The study adds to the available literature in that it was performed on a large sample of men and women with wide variation in both age (18 to 81 years) and BMI (from normal weight to class 3 obesity). However, it has several limitations. First, as it was performed in subjects enrolled at a nutritional research centre, its findings are unlikely to extend to the general population. Second, because of its cross-sectional nature, it cannot test whether changes in BE are associated with changes in body weight and a cohort study is needed to test this hypothesis. Third, we did not separate moderate from severe BE, as it is sometimes done using BES, as in our view this is more coherent with the screening psychometric properties of BES.

Our estimate of 17 % for the prevalence of BE is in agreement with recent studies^(^ ^14^ ^,^ ^15^ ^)^ but lower than that reported by other studies^(^ ^13^ ^,^ ^31^ ^)^. The most likely explanation for this difference is that we did not restrict our analysis to young obese women as done by most studies^(^ ^7^ ^,^ ^31^ ^,^ ^32^ ^)^. Including males, elders and normal-weight subjects, as we deliberately did here, is expected to decrease the overall prevalence of BE. Up to now, few studies have evaluated men^(^ ^5^ ^,^ ^14^ ^–^ ^17^ ^)^ and normal-weight subjects^(^ ^14^ ^,^ ^17^ ^)^. In order to allow a comparison with the available studies, performed mostly on obese women, the prevalence of BE in women in the present study was 14 % (95 % CI 12 %, 16 %) for normal-weight, 18 % (95 % CI 17 %, 20 %) for overweight, 24 % (95 % CI 22 %, 27 %) for class 1 obesity, 31 % (95 % CI 27, 35 %) for class 2 obesity and 32 % (95 % CI 26, 39 %) for class 3 obesity. On the other hand, in men we found a prevalence of BE of 1 % (95 % CI 1 %, 2 %), 5 % (95 % CI 3 %, 7 %), 10 % (95 % CI 8 %, 13 %), 12 % (95 % CI 8 %, 16 %), 17 % (95 % CI 9 %, 24 %) in normal-weight, overweight and 1, 2, 3 obesity classes, respectively. Our estimate of 17 % for the prevalence of BE is also lower than what we reported in a smaller sample of the same population (*n* 1472)^(^ ^33^ ^)^. Much of this difference stems from the fact that we defined BE as a BES score ≥18 instead of a BES score ≥17, as we did in our previous analysis. The reason for this choice is that, even though the BES≥17 cut-off point is used by most Italian studies, the BES≥18 cut-off point allows international comparisons^(^ ^8^ ^,^ ^34^ ^)^. It is noteworthy how a small change in the cut-off point used to diagnose BE can make its prevalence to vary so widely.

In agreement with other studies^(^ ^35^ ^,^ ^36^ ^)^, the prevalence of BE was higher in women than in men, decreased with increasing age and increased with increasing BMI. Although some studies did not detect between-sex differences in the prevalence of BE^(^ ^15^ ^,^ ^37^ ^)^, other studies reported a greater prevalence of BE among women^(^ ^5^ ^,^ ^33^ ^,^ ^34^ ^)^. Interestingly, there was virtually no change in the association of sex with BE after correction for confounders. In keeping with other studies^(^ ^7^ ^,^ ^31^ ^)^, we found that the prevalence of BE decreased with increasing age. The effect of age on BE prevalence was similar at univariable and multivariable analysis, with an increase of 1 year in age associated with decrease of 1 % in BE prevalence. As expected^(^ ^5^ ^,^ ^14^ ^)^, the prevalence of BE was directly associated with BMI. After correction for multiple confounders, an increase of 1 kg/m^2^ in BMI was associated with an increase of 5 % in BE prevalence. Very few data are presently available on the prevalence of BE in normal-weight subjects^(^ ^38^ ^,^ ^39^ ^)^. We found that BE was present also among normal-weight subjects, especially among women, although to a much lesser extent than among overweight and obese subjects.

Contrarily to other researchers^(^ ^14^ ^,^ ^15^ ^,^ ^40^ ^)^, we found that being married was a protective factor for BE. Other variables being equal, a married subject in our study had in fact a decrease of 12 % in BE prevalence. A direct association of BE with smoking was reported by other researchers^(^ ^37^ ^,^ ^41^ ^)^. In the present study, smoking was associated with a 15 % increase in the prevalence of BE. Interestingly, and contrarily to other researchers^(^ ^42^ ^)^, we found an inverse association between BE and physical activity. Being active was in fact associated with an 11 % lower prevalence of BE. In agreement with most^(^ ^13^ ^,^ ^14^ ^,^ ^17^ ^,^ ^31^ ^)^ but not all^(^ ^5^ ^,^ ^7^ ^)^ studies, we found that anxiety and depression were more prevalent in subjects with than in those without BE.

In conclusion, BE is common in individuals seeking to lose or maintain weight. The prevalence of BE is higher in young obese women. However, BE is present also in men, elders and normal-weight subjects. These findings reinforce the need to evaluate the eating behaviour of individuals starting weight loss or maintenance programmes.
